# Exome analysis reveals species divergence in *TYR* and identifies species genetic markers in five endemic *Macaca* species on Sulawesi Island

**DOI:** 10.1186/s12862-025-02407-6

**Published:** 2025-07-03

**Authors:** Xiaochan Yan, Nami Arakawa, Kanthi Arum Widayati, Laurentia Henrieta Permita Sari Purba, Fahri Fahri, Bambang Suryobroto, Yohey Terai, Hiroo Imai

**Affiliations:** 1 Molecular Biology Section, Center for the Evolutionary Origins of Human Behavior, Kyoto University, Inuyama, Japan; 2 Research Center for Integrative Evolutionary Science, SOKENDAI (The Graduate University for Advanced Studies), Hayama, Japan; 3 Department of Biology, IPB University, Bogor, Indonesia; 4 Department of Biology, Tadulako University, Palu, Indonesia; 5 Present Address: College of Bioresource Sciences, Nihon University, Fujisawa, Japan; 6 Present Address: Faculty of Biotechnology, Duta Wacana Christian University, Yogyakarta, Indonesia

**Keywords:** Interspecific, Coat color, Melanism, Tyrosinase, Fixation

## Abstract

**Background:**

One of the greatest challenges for evolutionary biologists is explaining the vast diversity observed in nature. On Sulawesi Island, macaque species (genus Macaca) have rapidly diverged from their common ancestor, displaying remarkable variability in body morphology and coat color. Despite low overall genetic variation among these macaques, limited hybridization occurs between neighboring species, possibly due to genomic divergence or local adaptations that act as barriers to interbreeding. This study aims to investigate highly divergent regions that might contribute to the distinct genetic and phenotypic characteristics differentiating the five Sulawesi macaque species. Additionally, it explores how these genetic differences influence biological functions, and identifies species-specific genetic markers for species identification and conservation.

**Results:**

Using whole exome sequencing of 46 individuals, approximately 550 highly divergent genes were identified across four pairwise species comparisons. Gene Ontology (GO) analysis revealed that these genes were enriched in critical biological processes, including cell adhesion, pigmentation, signal transduction, and stress responses. Among these, pigmentation-associated genes, such as *TYR* and *LRIT3*, exhibited highly divergent single nucleotide polymorphisms (SNPs). Missense mutations in *TYR* (D132N) and *LRIT3* (S394P, Y363D) were likely linked to the dark coat colors of *Macaca nigra* and *Macaca nigrescens*, highlighting their contribution to species-specific traits. Furthermore, hundreds of fixed SNPs were identified as potential species-specific markers for species discrimination, providing valuable resource for distinguishing Sulawesi macaque species.

**Conclusions:**

This study provides critical insights into the genetic mechanisms underlying species divergence and coat color variation in Sulawesi macaques. Highly divergent genomic regions between neighboring species likely contribute to species divergence and reinforce reproductive isolation. Enriched GO terms and pathways suggest that genetic divergence impacts key biological processes, including pigmentation, signal transduction, cell adhesion, and stress responses. Specifically, divergence in pigmentation-related genes such as *TYR* may play a role in interspecies differences in coat color, facilitating local adaptation, mate selection, and species identification. Additionally, the identification of species-specific genetic markers holds significant potential for conservation efforts, such as monitoring populations at risk of hybridization or genetic introgression. These findings advance our understanding of the genetic diversity in this unique primate group.

**Supplementary Information:**

The online version contains supplementary material available at 10.1186/s12862-025-02407-6.

## Introduction

Sulawesi Island, located in the Wallacea region, is the oldest and largest island in the archipelago. It harbors a remarkable diversity of endemic species, including the tarsier, mountain anoa, giant civet, and Sulawesi macaques [1, 2]. The Sulawesi macaques, which consists of seven species, inhabit distinct adjacent geographical regions on Sulawesi Island, with their ranges largely defined by allopatric boundaries [3, 4]. The Sulawesi macaques belong to the *silenus-sylvanus* group, one of three major phylogenetic groups described in the genus *Macaca*, alongside *M. sylvanus*,* M. silenuys*, and *M. nemestrina* [5]. It is hypothesized that an ancestral population closely related to *M. nemestrina* on Borneo dispersed to Sulawesi, leading to the evolution of seven endemic *Macaca* species on the Island [6].

Even though Sulawesi macaques are allopatrically distributed, presumable hybrids of Sulawesi macaques have been reported, except for *M. brunnescens*, which is confined to the southeastern islands. Although the six species of Sulawesi macaques exhibit distinct morphological differences, individuals displaying intermediate traits, likely representing hybrids, have been observed in the border zone of their respective habitats [7–12]. Gene flow between Sulawesi macaque species was initially inferred from these individuals with intermediate morphological characteristics [8, 13]. Later studies using microsatellite markers further supported the possibility of gene flow occurring near habitat boundaries [14]. Notably, the hybrid zones between pairs of Sulawesi macaque species are consistently located in stable and narrow regions, suggesting ongoing hybridization with limited introgression [13].

In the context of interspecific gene flow, genes that govern adaptive and reproductive traits specific to each species often act as barriers to gene flow [15]. Genomic regions that are highly divergent between two species experiencing gene flow, commonly referred to as gene flow barriers, have been shown to contain genes associated with local adaptation and reproductive isolation [15–22].

One of the most notable features of Sulawesi macaque is their coat color, which varies greatly in pattern and intensity between species [23]. Northern species, such as *M. nigra* and *M. nigrescens*, exhibit uniformly dark coats, whereas southern species, like *M. hecki*, display lighter pigmentation on their legs, forearms, and thighs [7]. In mammals, coat color is hypothesized to play critical roles in conspecific recognition, mate choice, and prevention of interbreeding [24]. Studies in rodents have shown a strong positive correlation between coat color and the background color of their environment, suggesting that natural selection has been acting on this trait [25]. Similarly, Sulawesi macaques have been observed to visually discriminate between closely related species, showing a longer looking time to conspecifics [26]. It is speculated that the remarkable divergence in coat color among these macaques may serve as an external signal to prevent interbreeding [27].

Based on these findings, we hypothesized that highly divergent genomic regions between neighboring species might act as barriers to prevent gene flow and maintain species integrity. If coat color serves as a barrier to gene flow, the genes responsible for coat color formation would exhibit high divergence between species and be located in these genomic regions. In this study, we aimed to identify highly divergent genomic regions among Sulawesi macaque species with adjacent distribution and assessed the relationship between genes within these regions and coat color variation. Furthermore, we sought to identify species-specific genetic markers for species identification and to contribute to the conservation of these endangered species in the future.

## Method

### Subject and sampling

**Fig. 1:**
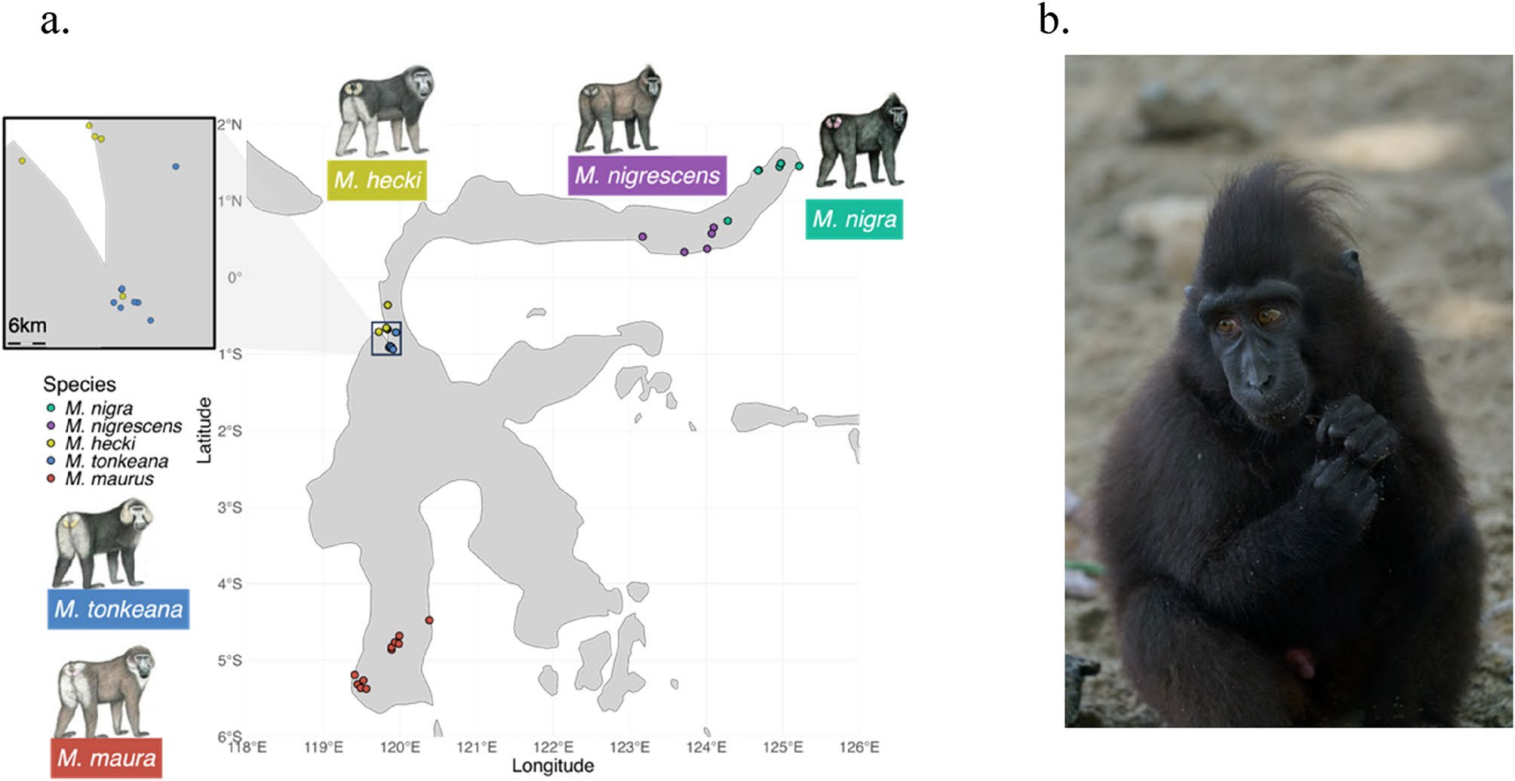
Fourty-six genetic samples were collected from monkeys in Sulawesi Island. The map (**a**) showed the approximate sampling location of samples. Latitude and Longtitude are available upon request to corresponding author. (**b**) showed a captive monkey of *M. nigra *in Sulawesi Island

Saliva samples were collected from 46 captive monkeys representing five species on Sulawesi Island: *M. maurus (N = 11),* *M. tonkeana (N = 11)*, *M. hecki (N = 9)*, *M. nigrescens (N = 6)*, *and M. nigra (N = 9)* (Fig. 1a). Detailed sample information is provided in Table S1. These samples were obtained from various locations that represent geographical ranges of each species. Following the methodology described by Watanabe et al. [12], we collected saliva samples from the monkeys and recorded information about their origins from the persons responsible to take care of the monkey. To evaluate the reliability of the information about the monkeys’ origins, we classified the “information fidelity” according to the following criteria: “A” the monkey was captured by the current owner or a member of their immediate family; “B” the monkey was transferred once from its original owner to the current owner; “C” the current owner could state the monkey’s origins with some certainty; and “D” the monkey may have been transferred between multiple owners. These criteria depended completely on the statements by the owners, so that there could be some mistakes even in criteria “B” or “C.” However, in most cases, we were able to obtain reliable information on the original sources of the monkeys. It is also important to note that the monkeys in captivity were unlikely to be related to one another since they were not being used for breeding.

Saliva was collected using cotton swabs rubbed inside the mouth, and samples were stored in 2-ml tubes containing lysis buffer (0.5% sodium dodecyl sulfate, 100 mM EDTA, 100 mM Tris-HCl, and 10 mM NaCl) following the protocol of Inoue et al. [28].

### Exome library construction

Genomic DNA was extracted from saliva samples using DNeasy Blood and Tissue Kits (Qiagen, Hilden, Germany). Concentration of double strain DNA (dsDNA) was determined by Qubit 2.0 fluorometer, using a high sensitivity Qubit Assays Kit. For preparing the DNA library, firstly, 5 ng genomic DNA was fragmented ultrasonically into 500 bp. The NEBNext Ultra II DNA Library Prep Kit for Illumina (New England Bio Labs, Ipswich, MA, USA) was employed to prepare and enrich the DNA library. Following the manufacturer’s instructions, the final concentration of the enriched, fragmented DNA was calculated using a bioanalyzer. Subsequently, the enriched DNA was hybridized with a human exome library using a SeqCap EZ Hybridization and Wash Kit (Roche, Germany) [29]. The SeqCap EZ Human Exome Library targets approximately 44 Mb of the human exonic regions. The SeqCap system utilizes 55- to 105-base DNA probes to capture known coding DNA sequences (CDS) from the NCBI Consensus CDS Database, RefSeq, and Sanger miRbase [30]. Paired-end (2 × 150 bp) sequencing was carried out using the HiSeq2500 platform. Each sample achieved high sequencing coverage (> 50×), producing more than 40 million raw reads with a read length of 150 bp, covering a total exome region of 44 Mb. Quality control procedures confirmed that the captured regions represent a reliable subset of expressed genes in *Macaca* species. These sequencing metrics, including number of raw reads and length, were provided in Supplementary Table 1 for reference.

### SNP calling and variant filtering

Single nucleotide polymorphisms (SNPs) and variants were called using the Genome Analysis Toolkit (GATK) version 4.1.6.0 [31]. Raw reads were trimmed to remove adaptor sequences and subsequently were aligned to the *Macaca mulatta* reference genome (assembly Mmul_10) using CLC Genomomic Workbench (version 20). Variants were identified using GATK’s HaplotypeCaller in GVCF mode for each sample and combined across all samples using CombineGVCFs. Joint genotyping was performed using GenotypeGVCFs to produce a multi-sample VCF file.

To retain high-confidence SNPs for downstream analysis, variant filtering was performed using VCFtools and BCFtools with the following criteria: Sites with a phred-scaled genotype quality (GQ) below 20 and a read depth (DP) ≤ 5 were excluded from the analysis [32]. SNPs with missing genotypes across any sample were excluded. No minor allele frequency (MAF) cutoff was applied.

### Annotation of divergent SNPs

Local adaptations can result in increased population differentiation, where high Fst values may indicate closely linked loci under selection, thereby identifying genes undergoing directional or linked to locally advantageous alleles. We employed an exome-wide sequencing approach to examine functional variation between four neighboring Sulawesi macaque species pairs: *M. nigra/M. nigrescens* (NgNc), *M. nigrescens/M. hecki* (NcH), *M. hecki/M. tonkeana* (HT), and *M. tonkeana/M. maurus* (TM).

Population divergence was assessed using Fst-based approaches, where population divergence at each SNP was calculated using Weir and Cockerham’s Fst, implemented in VCFtools. The threshold for Fst outliers was set at the top 5% of the Fst values, corresponding to the top 5% of the empirical distribution of all tested SNPs. To refine the analysis, a Fisher’s exact test was applied to identify non-overlapping 50,000-bp genomic windows enriched for significant SNPs, using an enrichment significance threshold of *p* < 10^*5*^. Genomic regions meeting this criterion were considered as candidate divergent regions. This method allows for the identification of genomic regions that exhibit substantial differentiation between populations. While SNPs within these windows may exhibit linkage disequilibrium, we do not assume complete independence between SNPs in this analysis. A Manhattan plot was generated to visualize the Fisher’s exact P-values across the genome, highlighting outlier SNPs and candidate regions indicative of species divergence.

SNPs annotation was retrieved from the *Macaca mulatta* genome assembly ver. 10 (Mmul_10), and genes containing the fixed SNPs were identified through the Ensembl Biomart tool [33]. To assess the potential functional impact of the identified genetic variants, each SNP was further analyzed using the Variant effect predictor (VEP) webtool [34]. This allowed us to categorize the SNPs according to their predicted functional consequences. The biological significance of these genes was explored through Gene Ontology(GO) enrichment analysis and Kyoto Encyclopedia of Genes and Genomes (KEGG) enrichment analysis using the Metascape platform (version 3.5) [35]. Functional enrichment was performed based on human orthologs of *Macaca* genes, given the high degree of coding sequence conservation between macaques and humans [36]. This strategy is consistent with standard practices in primate comparative genomics and leverages the extensive functional annotation available for the human genome [37]. For each given gene list, all annotated genes in the *Homo sapiens* genome were used as the background set for enrichment. Terms with *p-value* < 0.05, minimum count 2, and 1.5 < enrichment factor (enrichment factor is the ratio between observed count and the count expected by chance) are collected and grouped into clusters based on their membership similarities. The most statistically significant term within a cluster is chosen as the one representing the cluster. The top 20 significant enriched clusters were retained after applying Benjamini-Hochberg correction for multiple testing.

### Detection of species-specific markers

To identify species-specific genetic markers, we focused on fixed SNPs (Fst = 1), which indicate complete genetic differentiation, with alternate alleles fixed between populations. These SNPs serve as strong candidates for species-specific markers. For each fixed SNP, we determined its genomic location, predicted functional impact (e.g., synonymous, nonsynonymous, intronic), and associated gene annotation. We further examined the chromosomal and regional distribution of these SNPs, and highlighted those located in exonic regions, particularly those predicted to cause functional changes, such as missense variants. While the SNPs themselves are considered species-specific markers, the genes in which they reside may represent functionally relevant targets for further evolutionary or functional analysis.

### Analysis of *TYP* Exon 1 diversity and divergence

To investigate sequence diversity and genetic differentiation in the *TYP* exon 1 region among Sulawesi macaque species, we extracted aligned exon 1 sequences and calculated various diversity and divergence metrics using DnaSP v6 [38]. These included within-species diversity (Hs, Ks), between-species divergence (Kxy, Da, Dxy), and population differentiation indices (Fst, Gst, Nst, GammaSt, DeltaSt). Additionally, we calculated the nucleotide diversity for Chromosome 14 using non-overlapping 50,000-bp genomic windows.

## Result

### Fst distribution

**Fig. 2:**
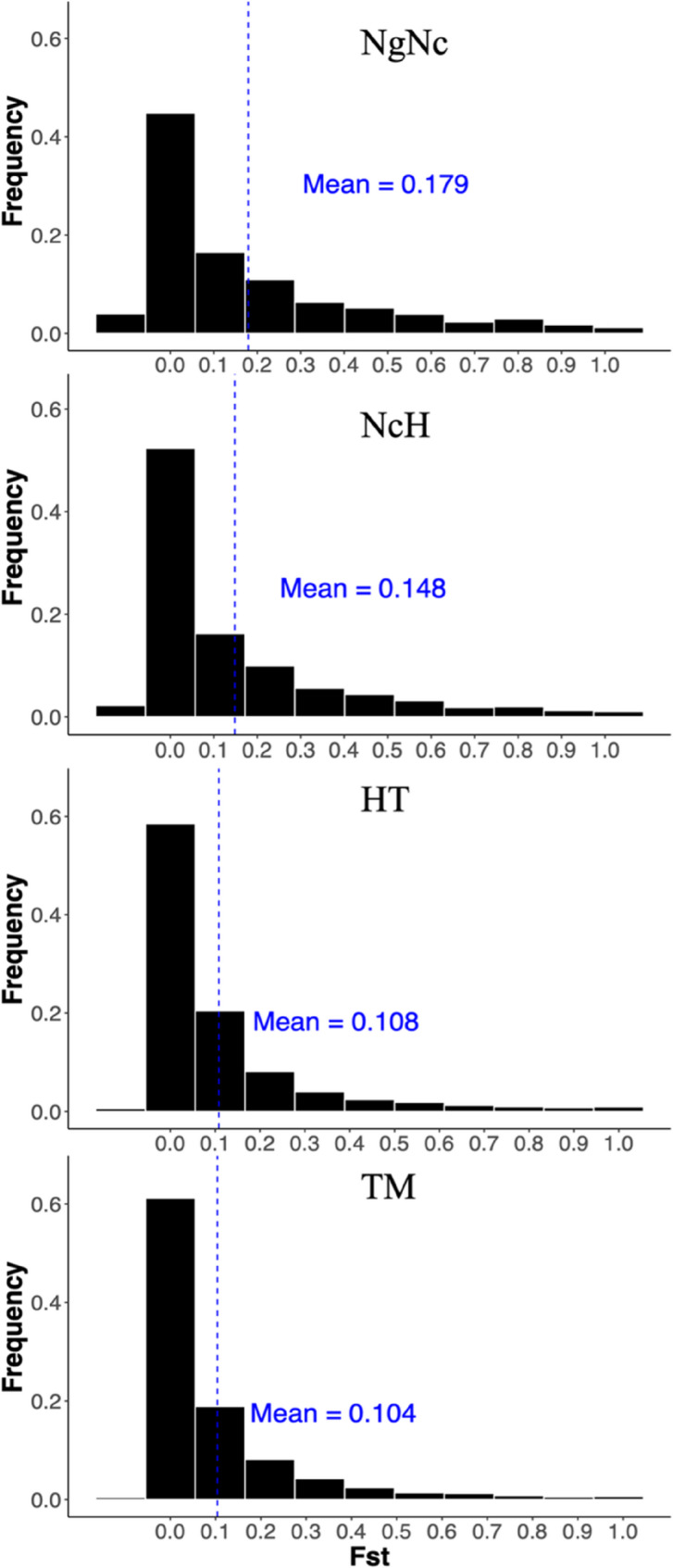
Distribution of Fst values in all four pairwise comparisons. Histogram showing Fst distribution between *M. nigra/M. nigrescens* (NgNc), *M. nigrescens/M. hecki* (NcH), *M. hecki/M. tonkeana* (HT) and *M. tonkeana/M. maurus* (TM). Fst distributions for all four comparisons were strongly skewed with approximately 40–60% of SNPs having an Fst under 0.05. Mean of Fst value was highlighted in blue

In total, we identified over 200,000 SNPs in each pairwise comparison of Sulawesi macaque species (Supplemental data). The total number of SNPs ranged from 215,435 in the comparison of NgNc to 411,879 in the comparison of TM (Table 1). Notably, the highest number of SNPs was detected in the comparison of TM, which showed relatively low genetic differentiation with a mean Fst of 0.104. The mean Fst values for other comparisons were 0.108 for HT and 0.148 for NcH. In contrast, the NgNc comparison exhibited higher genetic differentiation, with a mean Fst of 0.179. The Fst distributions for all four comparisons were strongly skewed, with approximately 40–60% of SNPs having an Fst below 0.05 (Fig. 2).

**Table 1:** An overview of the SNP calling results and the top 5% Fst value SNPs identified in the four pairwise comparisons: *M. nigra/M. nigrescens* (NgNc), *M. nigrescens/M. hecki* (NcH), *M. hecki/M. tonkeana* (HT) and *M. tonkeana/M. maurus* (TM)

**Comparisons**	**NgNc**	**NcH**	**HT**	**TM**
SNP Summary Information				
Total SNPs	216078	272817	400683	413023
CHROM SNPs	215435	272010	399578	411879
Top5% SNPs	10772	13601	19979	20594
Diverged SNP Composition Percentages				
intron_variant (%)	49.9	49.76	49.52	48.97
synonymous_variant (%)	19.9	19.92	19.84	20.1
missense_variant (%)	11.22	11.16	12.42	12.44
3_prime_UTR_variant (%)	2.32	2.12	2.08	2.08
downstream_gene_variant (%)	1.83	1.88	1.79	2.03
upstream_gene_variant (%)	1.32	1.4	1.23	1.26
intergenic_variant (%)	1.58	2.22	2.28	2.39
Others (%)	11.93	11.54	10.84	10.73
Diverged Exonic SNPs in windows				
Diverged SNPs in Exon	488	405	419	363
Diverged Genes	272	229	192	206
Diverged Nonsynonymous SNPs	174	155	156	129

### Identification of highly divergent genomic regions

**Fig. 3:**
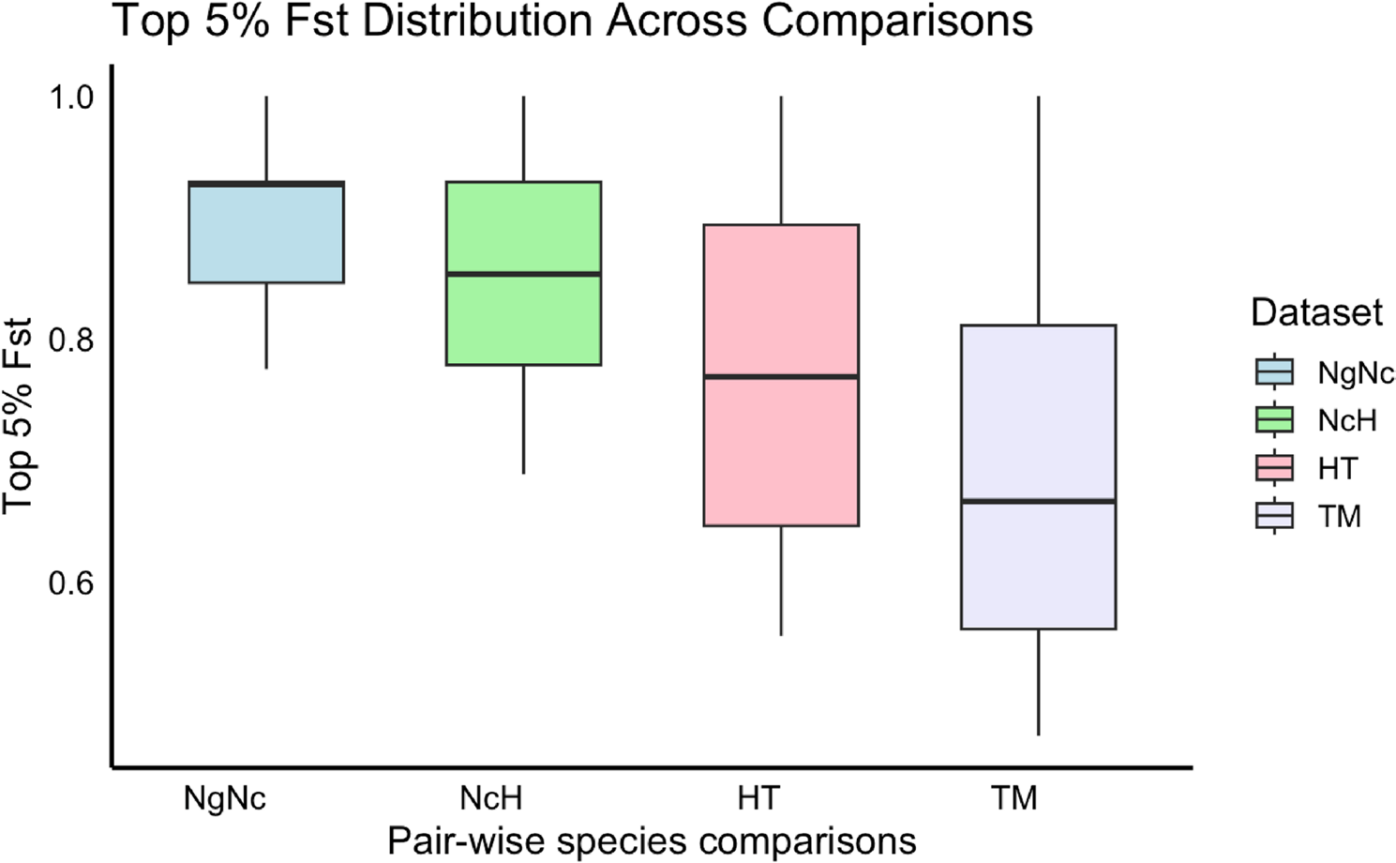
The distribution of the top 5% SNPs Fst values across all four pairwise species comparisons: *M. nigra/M. nigrescens* (NgNc), *M. nigrescens/M. hecki* (NcH), *M. hecki/M. tonkeana* (HT) and *M. tonkeana/M. maurus* (TM)

To determine the genomic regions under potential selection, we identified putative targets enriched with the SNPs within the top 5% of Fst value in each comparison. The mean Fst values for the top 5% SNPs in comparisons were 0.899 (SD = 0.074) in NgNc, 0.857 (SD = 0.100) in NcH, 0.771 (SD = 0.138) in HT, and 0.699 (SD = 0.162) in TM, respectively. Furthermore, candidate regions were identified using Fisher’s exact test with a significance threshold of *P* < 10^*−5*^ (Fig. 3a), resulted in 272, 229, 192, 206 candidate genes in NgNc, NcH, HT, and TM, respectively. Approximately 31.41–32.94% of these highly divergent SNPs were located in exonic regions. In total, 550 missense SNPs were identified across four comparisons (Table S2).

### Functional enrichment of candidate genes

To explore the possible functional implications of the candidate genes, we performed gene ontology (GO) and pathways enrichment analysis. The top 20 most significantly enriched clusters for each comparison are summarized in Table S3. In NgNc, overrepresented GO terms included “mRNA processing” and “homophilic cell adhesion via plasma membrane adhesion molecules,” while pathways such as “Signaling by Rho GTPases” and “Viral Infection Pathways” were enriched. Similarly, NcH candidate genes were enriched in pathways like “TGF-beta signaling pathway” and GO terms including “intracellular protein transport” and “mitotic cell cycle process.” Notably, 26 genes (*HMGCR*,* HYAL1*,* PPP1CC*,* ROM1*,* TP53*,* TYR*,* HYAL3*,* MAP4K3*,* SLC24A1*,* TIPIN*,* LRIT3*,* CD44*,* NABP1*,* KDM5B*,* CD14*,* CYP1B1*,* STAT1*,* B4GALT3*,* B4GAT1*,* GALK1*,* NT5C2*,* SAMHD1*,* AAAS*,* PIK3CA*,* GANAB*,* PGGHG*) in NcH were associated with “response to light stimulus,” with six them (*HYAL1*,* TP53*,* TYR*,* HYAL3*,* MAP4K3*,* TIPIN*) further linked to “response to UV,” highlighting their role in pigmentation regulation.

### Variants in *TYR* and pigmentation genes

**Fig. 4:**
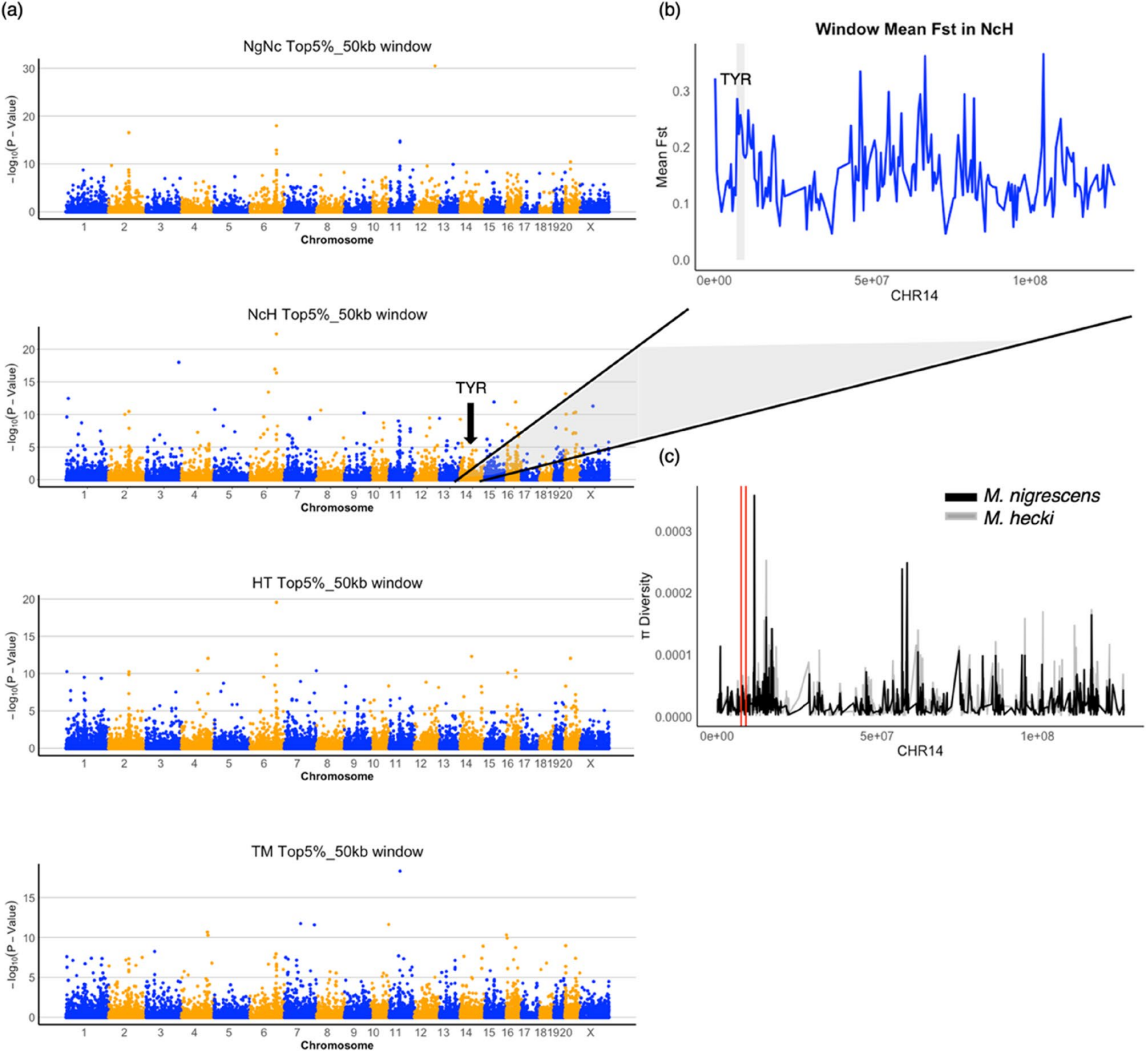
Collapsing analysis identifies *TYR* as a highly divergent gene. **a** Manhattan plots of the population differentiation in 50-kb nonoverlapping windows across all four pairwise species comparisons: *M. nigra/M. nigrescens* (NgNc), *M. nigrescens/M. hecki* (NcH), *M. hecki/M. tonkeana* (HT) and *M. tonkeana/M. maurus* (TM). **b** Manhattan plot of 50-kb mean Fst across Chromosome 14 in NcH comparison. The dashed block highlights the neighboring windows and *TYR* window. **c** Nucleotide diversity for 50-kb nonoverlapping windows across Chromosome 14 in *M. nigrescens* and *M. hecki*. The red lines highlight the neighboring windows and *TYR* window

Notably, the *TYR* (Tyrosinase) gene, known for its critical role in triggering the first and rate-limiting step in melanin biosynthesis, exhibited significant genetic variation in Sulawesi macaques. Among the top5% SNP, we detected 10 variants located in TYR, comprising 5 synonymous SNPs, 3 intron SNPs and 2 missense SNPs. The genomic windows containing the *TYR* gene showed high level of differentiation in the NcH comparison (Fig. 4b) and exhibited low nucleotide diversity in both *M. nigrescens* and *M. hecki* (Fig. 4c). Both analyses were performed using non-overlapping 50-kb windows.

**Fig. 5:**
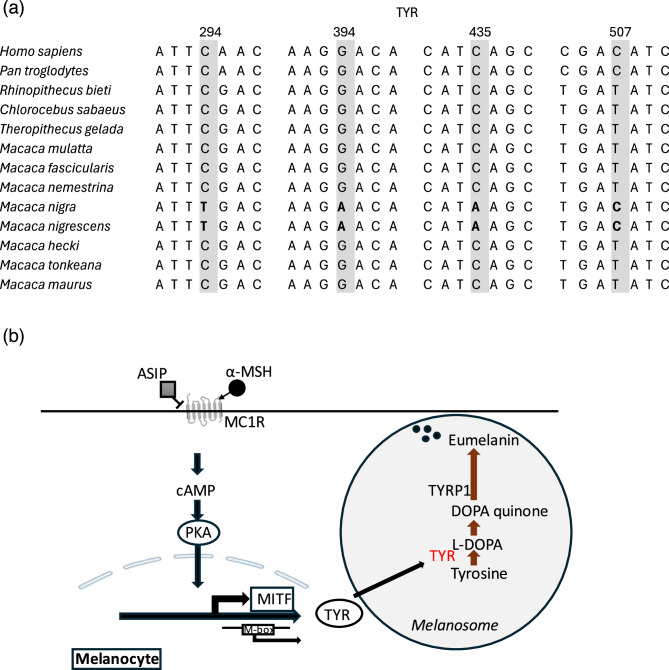
Divergent sites in *TYR* and its role in the melanogenesis pathway. **a** Highly divergent SNPs (top 5% Fst) located in the first exon of TYR across primate species. **b** Schematic graph of TYR’s role in the melanogenesis pathway

Notably, four of these SNPs were located in the first exon (Table 2). Furthermore, these four SNPs were shared between *M. nigrescens* and *M. nigra*, distinguishing them from the other Sulawesi macaque species (Fig. 5a).

**Table 2:** Diverged SNPs in *TYR* gene in all four comparisons. The values represent site-specific Fst estimates for the comparisons*: M. nigra/M. nigrescens* (NgNc), *M. nigrescens/M. hecki* (NcH), *M. hecki/M. tonkeana* (HT) *and M. tonkeana/M. maurus* (TM). Each row represents a SNP detected as top5% Fst value in one or more comparisons. SNP: single nucleotide polymorphism

SNP	NgNc	NcH	HT	TM	Consequence	Impact	Symbol	Exon	CDS_position	Protein_position	Amino_acids	Codons	BLOSUM62
1	0.0000	1.0000	0.0000	0.0000	synonymous_variant	LOW	TYR	1/5	294	98	F	ttC/ttT	-
2	0.0026	0.8535	0.0000	0.0000	missense_variant	MODERATE	TYR	1/5	394	132	D/N	Gac/Aac	1
3	0.0000	1.0000	0.0000	0.0000	synonymous_variant	LOW	TYR	1/5	435	145	I	atC/atA	-
4	0.0000	1.0000	0.0000	0.0000	synonymous_variant	LOW	TYR	1/5	507	169	D	gaT/gaC	-
5	0.0026	0.8535	0.0000	0.0000	intron_variant	LOW	TYR	-	-	-	-	-	-
6	0.0000	0.0000	0.1843	0.5615	intron_variant	MODIFIER	TYR	-	-	-	-	-	-
7	0.0000	0.0000	0.0000	0.7188	intron_variant	MODIFIER	TYR	-	-	-	-	-	-
8	0.0000	0.1048	0.0000	0.4818	synonymous_variant	LOW	TYR	2/5	951	317	D	gaC/gaT	-
9	0.0000	0.8607	0.5354	0.5759	missense_variant	MODERATE	TYR	2/5	1003	335	A/T	Gct/Act	0
10	0.0000	0.9293	0.8325	0.0500	synonymous_variant	LOW	TYR	3/5	1146	382	N	aaC/aaT	-

Population differentiation analysis focusing on the first exon of *TYP *revealed that low Fst (0.12) and Dxy (0.0014) between *M. nigra* and *M. nigrescens*, contrasting with significantly higher Fst (average 0.89) and Dxy (average 0.0054) values between *M. nigra* and other Sulawesi macaque species (Table S4). Additional pigmentation-related genes, such as *ASIP* (Agouti-signaling protein) and *HPS*5 (Hermansky-pudlak syndrome 5), also exhibited missense variants. In *M. nigrescens*, a missense variant (E27G) in ASIP, which modulates melanin synthesis, was detected at a highly conserved site. Furthermore, a missense variant (S669L) in HPS5, involved in melanosome trafficking, was identified in *M. nigrescens* and at low frequencies in *M. nigra*. These variants may influence pigmentation by altering melanin production or transport mechanisms.

### Species-specific genetic marker

We next focused on fixed SNPs, defined as loci with an Fst value of 1 in each paired comparison. Each pairwise comparison contained numerous fixed SNPs, distributed across all chromosomes (Figure S1), with proportions ranging from 0.38 to 1.20% of the total SNPs identified. Although a human exome capture kit was used, a notable fraction of fixed SNPs were located outside annotated coding regions. Among the fixed SNPs, 41.18–45.00% were found in exonic regions. Notably, we identified significantly more fixed SNPs in the NgNc and NcH comparisons than in the other two pairwise comparisons.

For the NgNc comparison, we identified 2,598 fixed SNPs, of which 1,114 SNPs were located within the exons of 705 genes, comprising 306 missense SNPs. Similarly, in the NcH comparison, 2,788 fixed SNPs were found, with 1,226 exonic SNPs spanning 861 genes, including 347 missense SNPs. In the HT comparison, 1,870 fixed SNPs were detected, with 770 exonic SNPs spanning 559 genes, including 258 missense SNPs. Finally, the TM comparison yielded 1,580 fixed SNPs, with 711 exonic SNPs across 516 genes, comprising 225 missense SNPs.

### Genes with fixed SNPs across all four comparisons

We identified 11 genes containing fixed SNPs in all four comparisons, suggesting these genes might serve as species markers among Sulawesi macaques (Figure S2). Upon annotating the SNPs positions in these 11 genes, we identified a total of 54 fixed SNPs located on chromosome 2, 4, 6, 14, 16, 20, and X (Table S5). Among these, three variants were located in non-protein coding regions (5’ and 3’ UTR regions) in B4GAT1 (Beta-1,4-Glucuronyltransferase 1), KDM2A (Lysine Demethylase 2 A), and NHS (Nance-Horan Syndrome Protein) genes. Additionally, we detected a 9 bp deletion (GAFCAFCCC/EQP) variant in *ZFHX3* (Zinc Finger Homeobox 3) gene specific to *M. maurus*.

Given the hypothesis that missense variants are rare but can have significant functional consequences, we further concentrated on genes containing fixed missense variants to assess their potential impact on functional divergence among species. The genes analyzed included *TGM4* (Transglutaminase 4, missense SNPs, *N* = 2), *MC1R* (Melanocortin 1 Receptor, *N* = 4), NHS (*N* = 2), *BCOR* (BCL6 Corepressor, *N* = 1), and *RTL9* (Retrotransposon Gag Like 9, *N* = 4). *TGM4*, known for its role in male reproduction, harbored two missense variants (V168M, Y176H), both of which were predicted to have neutral effects based on PROVEAN analysis. Three genes, *BCOR*,* NHS* and *RTL9*, were in X chromosome and contained missense variants. *BCOR* encodes an epigenetic regulator involved in cell differentiation and body structure development. A missense variant, S613T, which involves a substitution of serine to threonine in exon 4 of *BCOR*, appears to have a neutral effect on protein function.

*NHS*, which encodes a Nance-Horan syndrome protein, may play a role in the development of eyes, teeth, and brain. We identified two missense variants located in exon 6 of *NHS*: A854T (substituting a hydrophobic residue with a polar residue) and V1294A (replacing valine with alanine). Both variants are predicted to have a neutral effect on protein function. *RTL9* (Retrotransposon gag-like protein 9, also referred to as *RGAG1*) is a neofunctionalized retrotransposon primarily expressed in the human testis, although its specific function remains unclear. We detected four missense variants in *RTL9* (A197D, S654A, T1015I and G1077C), all located in the first exon. Notably, the T1015I variant may potentially alter functional characteristics, although further experimental validation is needed.

*MC1R* encodes a receptor crucial for regulating the type and density of melanin synthesis. Four missense variants were identified in *MC1R*. The I293V variant appears to have a neutral effect, whereas the substitutions in conservative sites (H153P, C267Y, and E304G) are likely to induce significantly functional changes. These missense SNPs in *MC1R* could alter the functional properties of the receptor, potentially affecting melanin deposition in the macaques’ coat.

## Discussion

We identified highly divergent genomic regions between neighboring species, which were likely to contribute to species divergence and reinforcement to prevent interbreeding. The enriched GO terms and pathways suggested that high divergence among Sulawesi macaque species were involved in critical biological processes, including pigmentation, signal transduction, cell adhesion, and stress responses. Divergence in the pigmentation-related gene *TYR* may contribute to interspecies differences in coat color, potentially playing a role in local adaptation, mate selection and species identification [39, 40]. The missense mutation D132N in *TYR*, found exclusively in dark-coated species (*M. nigra* and *M. nigrescens*), suggests its potential role in melanin biosynthesis and species-specific pigmentation patterns. Furthermore, we identified a list of fixed SNPs that could serve as potential species-specific markers for species discrimination. These markers can assist in designing conservation strategies by identifying populations at risk of hybridization or genetic introgression in the future.

The *TYR* gene, encoding tyrosinase, plays a pivotal role in melanin biosynthesis, catalyzing the rate-limiting step of converting tyrosine to dopaquinone [41] (Fig. 5b). The expression pattern of TYR is restricted to neural crest-derived melanocytes and retinal pigment epithelial (RPE) cells. TYR variants potentially contribute to coat color variation through various mechanisms affecting melanin production, regulation, and intracellular transport within melanocytes [42]. In this study, *TYR* variants, particularly the missense mutation D132N, was shared exclusively by uniform dark-coated species (*M. nigra* and *M. nigrescens*). The low Fst values between *M. nigra* and *M. nigrescens* (0.12) and significantly higher Fst values with other Sulawesi macaques (0.89) further suggested the role of *TYR* in species divergence. In addition to *TYR*, we identified *LRIT3* (Leucine-Rich Repeat, Immunoglobulin-like and Transmembrane Domains 3) as another gene harboring highly divergent missense variants shared by *M. nigra* and *M. nigrescens*. While LRIT3 is primarily associated with transmission of a light-evoked stimulus from the cone photoreceptor cells to retinal bipolar cells [43], its potential role in facilitating FGFR1 (Fibroblast Growth Factor Receptor 1) trafficking from the endoplasmic reticulum to the Golgi apparatus suggests its potential role in pigmentation regulation [44]. FGFR signaling has been implicated in melanocyte development and melanin production, making LRIT3 a plausible regulator of pigmentation pathways. Two missense variants Y363D and S394P (Table S2), specific to uniform dark species, may influence FGFR1 activity, further effect melanin biosynthesis. These variants potentially contribute to coat color variation through various mechanisms affecting melanin production, regulation, and intracellular transport within melanocytes.

We identified fixed SNPs in *MC1R* that serve as species-specific genetic markers across all five macaque species. In our previous study, we confirmed that the species-specific variants in *MC1R* influenced its activity in both the presence and absence of ligands among Sulawesi macaques [45]. However, *MC1R* alone does not fully explain the pigmentation patterns observed in Sulawesi macaques. The *ASIP* gene, which acts as an antagonist to *MC1R* by promoting the synthesis of pheomelanin, presents another plausible candidate for pigmentation differences. A missense variant (E27G), identified in *M. nigrescens*, occurs at a highly conserved site in *ASIP*, potentially contributing to species-specific pigmentation differences. Since *M. nigrescens* lacks the agouti-banded hair pattern, it is hypothesized that reduced ASIP levels lead to lower pheomelanin production, contributing to its dark coat color. Interestingly, the E27G variant was also found in one individual of *M. nigra*. This raises questions about the specific role of the E27G variant in determining the dark coat. Further investigation is needed to determine whether the E27G variant contributes to the dark coat color in *M. nigrescens*. It appears that changes in ASIP regulation, rather than alterations in the coding sequence, may have a more significant influence in pigmentation in human [46]. This trend is also observed in other species, such as mice and dogs [47–51].

Despite these findings, several limitations remain. While missense variants such as D132N in TYR, Y363D and S394P in LRIT3 were identified, their functional roles in pigmentation and potential interactions with *FGFR* signaling require further validation. Additionally, phenotypic variation often arises through gene regulatory divergence, suggesting that differences in gene expression may play a significant role in coat coloration variation. Addressing these gaps in future research will offer a more comprehensive understanding of the genetic mechanisms underlying pigmentation and their contribution to the evolutionary dynamics and ecological adaptation of Sulawesi macaques.

Divergence in coat color may function as an external cue for species recognition, helping to prevent interbreeding and thereby promoting reproductive isolation through mate choice [27]. In small, isolated populations, however, coat color variation can also arise and become fixed through genetic drift, independent of selective pressures. When such populations come to contact, positive assortative mating based on coloration may further reinforce both genetic and phenotypic divergence. *Macaca nigra*, a critically endangered and highly specialized species, exhibits low nucleotide diversity (π = 0.0001), which might limit its adaptive potential and increasing susceptibility to genetic drift. Restricted gene flow may further contribute to coat color divergence, making drift a significant evolutionary force. While local adaptation cannot be ruled out, the combined effects of drift and non-random mating likely shape coat color variation in Sulawesi macaques. Future studies integrating genomic and ecological data are needed to clarify these dynamics, while conservation efforts should prioritize maintaining genetic diversity to support species viability.

A limitation of this study is the use of captive macaque samples for whole exome sequencing. While these samples were selected to capture a broad genetic range, they may not fully represent the genetic diversity of wild populations. Wild macaques may possess unique genetic variations not present in captivity, potentially limiting the generalizability of our findings. Future research incorporating wild populations is needed to enhance the comprehensiveness of the study.

## Conclusion

This study utilized whole-exome sequencing data from 46 individuals to investigate divergent genomic regions among five endemic *Macaca* species on Sulawesi Island. Approximately 550 genes were identified as highly divergent in at least one pairwise species comparison, with the observed differentiation potentially driven by positive selection or background selection acting on these regions. Notably, pigmentation-related genes such as *TYR* and *LRIT3* were enriched in these regions, underscoring their role in species differentiation. Furthermore, the identification of fixed SNPs as species-specific genetic markers offers valuable resources for species identification and conservation efforts. These markers can aid in monitoring populations at risk of hybridization or genetic introgression, supporting the development of strategies to maintain the genetic integrity of these endangered species. These findings deepen our understanding of genetic diversity and lay a foundation for future research on the ecological and evolutionary dynamics shaping primate species.

## Supplementary Information

Supplementary Material 1.

Supplementary Material 2.

## Data Availability

The data underlying this article has been submitted as a Bioproject and is available under Bioproject ID PRJDB13948.
