# Supplementary material for: Exome analysis reveals species divergence in TYR and identifies species genetic markers in five endemic Macaca species on Sulawesi Island
Source: BMC Ecol Evol. 2025 Jul 3;25:66. doi: 10.1186/s12862-025-02407-6 (PMC12225481; doi:10.1186/s12862-025-02407-6)

Supplementary Figure 1 Distribution of exonic fixed SNPs in genes and chromosomes. Gray bar represents the number of fixed SNPs locating in exon; blue bar represents the number of genes contained above differentiated SNPs. Fixed SNPs were distribution over all 20 auto chromosomes and sex chromosome X in all four pairwise species comparisons.

Supplementary Figure 2 Venn diagram of differentiated genes between pairwise species comparisons. *M. nigra/M. nigrescens* (NgNc), *M. nigrescens/M. hecki* (NcH), *M. hecki/M. tonkeana* (HT) and *M. tonkeana/M. maurus* (TM). The number of terms overlapped between comparisons are shown in the Venn diagram. Hundres genes contained differentiated SNPs were specific in each comparison. Eleven genes were commonly differentiated in four comparisons.


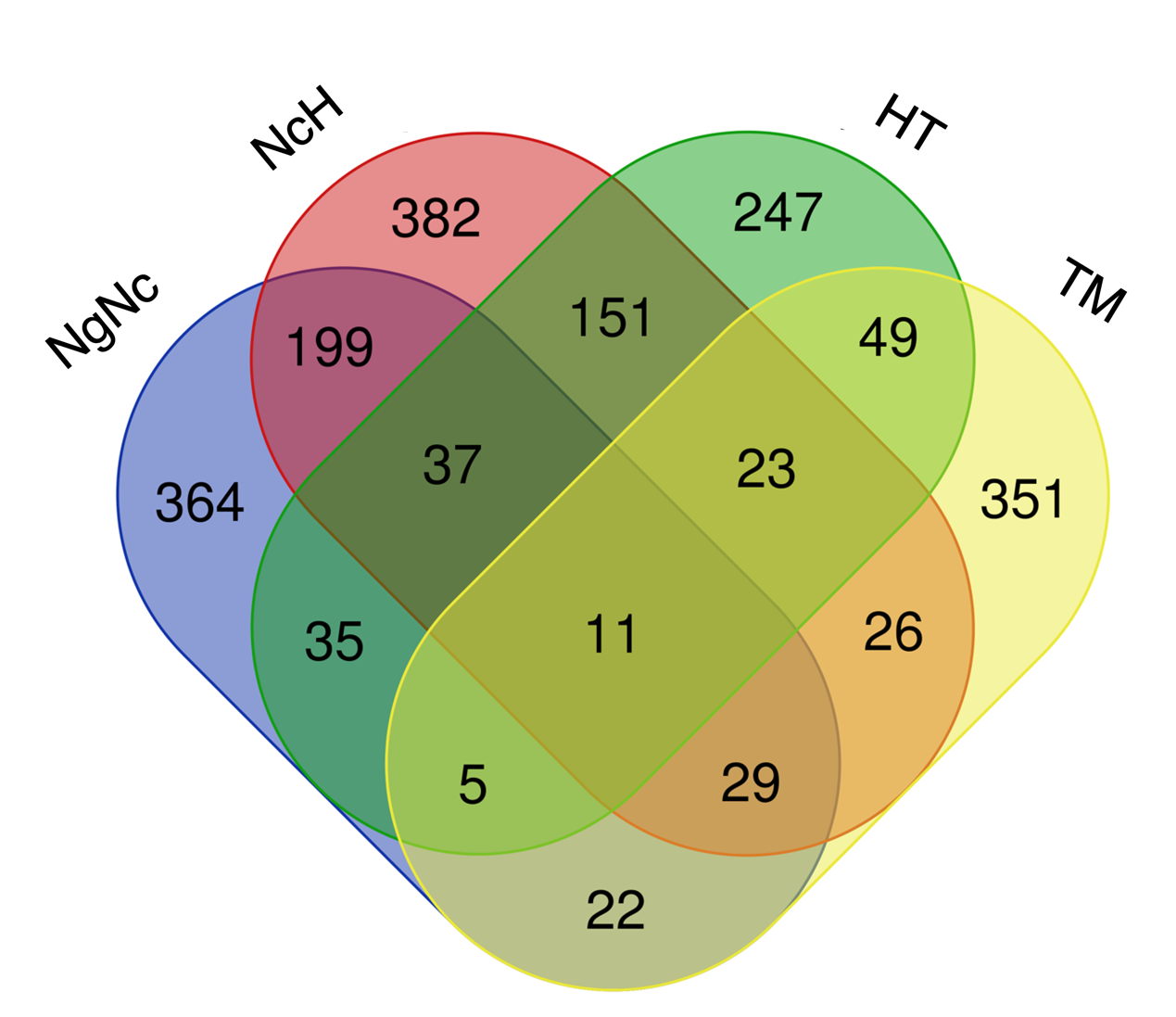

Supplement: Supplementary file 1 — Supplementary Material 1. [file 12862_2025_2407_MOESM1_ESM.docx]
